# Properties of a Laser Shock Wave in Al-Cu Alloy under Elevated Temperatures: A Molecular Dynamics Simulation Study

**DOI:** 10.3390/ma10010073

**Published:** 2017-01-18

**Authors:** Xiankai Meng, Jianzhong Zhou, Shu Huang, Chun Su, Jie Sheng

**Affiliations:** 1 School of Mechanical Engineering, Jiangsu University, Zhenjiang 212013, China; xiankaimeng@gmail.com (X.M.); huangs@ujs.edu.cn (S.H.); suc@czu.cn (C.S.); shengjie@ujs.edu.cn (J.S.); 2 School of Mechanical & Vehicle Engineering, Changzhou Institute of Technology, Changzhou 213022, China

**Keywords:** laser shock wave, molecular dynamics simulation, elevated temperature, Al-Cu alloy, dislocations

## Abstract

The laser shock wave (LSW) generated by the interaction between a laser and a material has been widely used in laser manufacturing, such as laser shock peening and laser shock forming. However, due to the high strain rate, the propagation of LSW in materials, especially LSW at elevated temperatures, is difficult to study through experimental methods. A molecular dynamics simulation was used in this study to investigate the propagation of LSW in an Al-Cu alloy. The Hugoniot relations of LSW were obtained at different temperatures and the effects of elevated temperatures on shock velocity and shock pressure were analyzed. Then the elastic and plastic wave of the LSW was researched. Finally, the evolution of dislocations induced by LSW and its mechanism under elevated temperatures was explored. The results indicate that the shock velocity and shock pressure induced by LSW both decrease with the increasing temperatures. Moreover, the velocity of elastic wave and plastic wave both decrease with the increasing treatment temperature, while their difference decreases as the temperature increases. Moreover, the dislocation atoms increases with the increasing temperatures before 2 ps, while it decreases with the increasing temperatures after 2 ps. The reason for the results is related to the formation and evolution of extended dislocations.

## 1. Introduction

A laser shock wave (LSW) is generated by the interaction between a laser and a material, and it has been widely used in laser manufacturing areas, such as laser shock peening [1], laser shock forming [2], and laser shock welding [3], etc. As one of the most important applications, laser shock peening (or laser peening) can generate high compressive residual stress and high yield strength by severe plastic deformation, which can significantly improve the fatigue life of materials [4,5,6,7]. Therefore, laser shock peening has become the most commonly used technology to extend the lifetime of aeronautical materials [8,9]. Al-Cu alloys are one of the most widely used aluminum alloys in aerospace industries. For example, 2024 aluminum is widely applied in the manufacture of aircraft engine blades or aircraft skins and, thus, it usually serves under severe mechanical loading conditions, in particular suffering fatigue damage [10]. Therefore, laser shock peening technology has been widely used to enhance the fatigue life of 2024 aluminum [11,12].

In order to increase the fatigue improvement generated by laser peening, a thermal-mechanical technology, warm laser peening, was developed by Ye in the early 2000s [13]. By conducting laser peening at elevated temperatures, warm laser peening has been proved to be an effective technology to increase the fatigue life of AISI4140 steel, and 6061 and 7075 aluminum alloy [14,15,16]. It also shows that the effects on materials’ properties significantly depend on the microstructures generated by warm laser peening, such as dislocations and precipitates. However, because of the high pressure and short pulse-width, the strain rate induced by LSW is extremely high. Thus, it is very difficult to explore the propagation of the LSW and the evolution of microstructures induced by the LSW through experimental methods. Therefore, the research about the LSW propagated in an Al-Cu alloy, especially the LSW under elevated temperatures, is still insufficient.

With the rapid development of computer hardware, molecular dynamics simulation has become an effective research tool to study the evolution of microstructures, such as dislocations and precipitates [17,18]. Bringa [19] demonstrated the Rankine-Hugoniot relations during shock processes through molecular dynamics simulation in single-crystal copper. Moreover, the propagation of a shock wave was simulated by different potential functions. It was indicated that embedded atom (EAM) potential was more suitable for the investigation of the shock wave propagating in metals. The molecular dynamics simulation provides a helpful method to explore the plastic deformation mechanism of laser peening and warm laser peening.

On the basis of the Al-Cu molecular dynamic model, the propagation of the LSW in an Al-Cu alloy was simulated by Lammps software (Version 24 Jan 2013, Sandia National Laboratories, Albuquerque, NM, USA) [20,21] in this study. The effects of elevated temperatures on the propagation of the LSW were researched. Moreover, the evolution of dislocations induced by the LSW was obtained and its mechanism under elevated temperatures was explored.

## 2. Materials and Methods

### 2.1. Laser Shock Wave (LSW)

**Figure 1:**
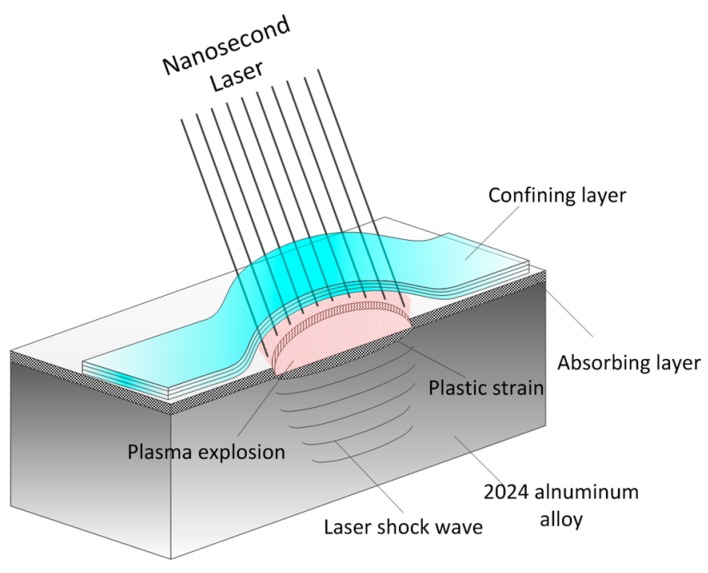
Schematic diagram of laser shock wave.

The schematic diagram of the LSW is shown in Figure 1. It can be seen that a plasma explosion is generated by the interaction of a nanosecond laser with an absorbing layer. Then the high pressure will be induced by the growing plasma explosion under the constraint of the confining layer and, thus, a shock wave will propagate in 2024 aluminum alloy with the reaction force of the plasma explosion. The confining layer and absorbing layer are used to increase laser shock pressure [22]. The absorbing layer, such as black paint and aluminum foil, is covered on the surface of the substrate to increase laser absorption and, thus, enhance laser shock pressure. Moreover, the absorbing layer can also protect the substrate from laser ablation. The confining layer, such as water and glasses, is covered on the surface of absorbing layer to constrain the plasma’s pressure and, thus, increase the laser shock pressure.

### 2.2. Molecular Dynamics Model and Simulation Methods

**Figure 2:**
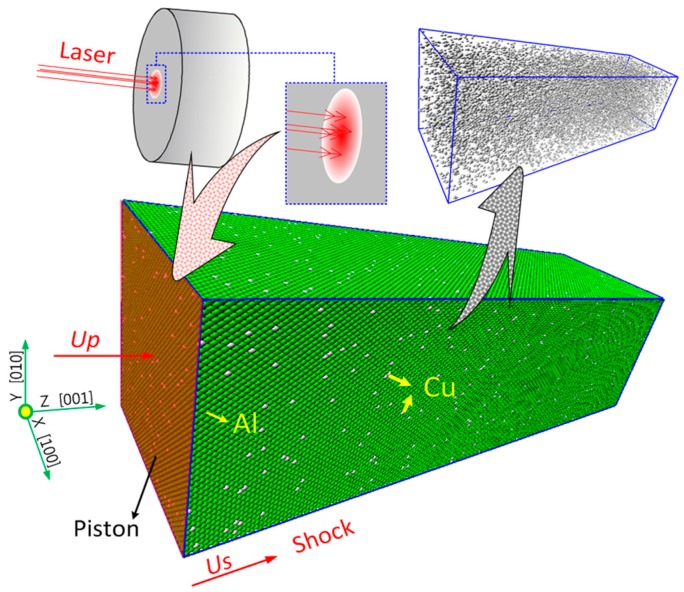
Molecular dynamics model of the Al-Cu alloy.

The molecular dynamic model of the Al-Cu alloy in this study is shown in Figure 2. Al-Cu alloy is one of the common materials with face-centered cubic (FCC) lattices and its lattice constant is about 4.05 Å [23]. In this paper, the dimensions of the Al-Cu model are *X* × *Y* × *Z* = 162 Å × 162 Å × 405 Å. Based on the composition of 2024 aluminum, the total number of atoms in this model is 640,000, and there are about 16,640 Cu atoms in this model. In order to guarantee the uniform distribution of Cu atoms in Al-Cu alloy, the Al-Cu alloy was constructed by the following procedures. First, a bulk of single-crystal Al was established using FCC lattices. Then, a certain number of Al lattices were selected randomly and one or two Al atoms in every selected lattice were replaced by Cu atoms. Finally, the Al-Cu alloy was obtained after relaxation.

Non-periodic boundaries were used in the shock direction (*Z* direction), while periodic boundaries were utilized in other directions (*X* and *Y* directions). In this study, a mixed EAM potential of Al-Cu alloy was used which contains the interactions between Al-Al atoms, Al-Cu atoms, and Cu-Cu atoms. The EAM potential is used in the Al-Cu alloy [24]:

(1) $$E=\frac{1}{2}\underset{j\neq 1}{\sum }\Phi_{ij}\left(\gamma_{ij}\right)+\underset{i}{\sum }F_{i}\left(\rho_{i}\right)$$

where Φ*_ij_*(γ*_ij_*) is the pair interatomic potential, *F_i_*(ρ*_i_*) is the embedding energy function, ρ*_i_* is the atomic density around an isolated atom, and γ*_ij_* is the distance between the *i*th atom and the *j*th atom.

A piston with a particle velocity *U*_p_ was used to generate the LSW in this study, as shown in Figure 2. The relation between the laser energy and the particle velocity can be deduced as follows: based on the research of Ballard [22], the plastic strain ε induced by laser shock wave can be written as:

(2) $$\varepsilon =\frac{2HEL}{3\lambda +2G}\left(\frac{P_{max}}{HEL}-1\right),$$

(3) $$P_{max}=0.01Z^{\frac{1}{2}}{\left(\frac{\alpha }{2\alpha +3}\right)}^{\frac{1}{2}}{\left(\frac{4E}{\pi R^{2}\tau }\right)}^{\frac{1}{2}},$$

where *HEL* is the Hugoniot elastic limit, λ is the Lame constant, *G* is the shear modulus, *P_max_* is the peak pressure induced by laser shock wave, *Z* is the reduced shock impedance between the target material and the confining medium, α is the portion of absorbed energy contributing to the thermal energy of the plasma, *E* is the laser energy, *R* is the radius of laser spot, and τ is the laser pulse width. Based on the propagation process of shock wave, the plastic strain ε can be written as:

(4) $$\varepsilon =\frac{U_{p}dt}{L}\;,$$

where *dt* is the time interval, *L* is the length of model in shock direction, *U*_p_ is particle velocity. Therefore, the relationship between particle velocity and laser energy can be seen as:

$$U_{p}=\frac{2HEL}{3\lambda +2G}\left(\frac{P_{max}}{HEL}-1\right)\frac{L}{dt}$$

(5) $$P_{max}=0.01Z^{\frac{1}{2}}{\left(\frac{\alpha }{2\alpha +3}\right)}^{\frac{1}{2}}{\left(\frac{4E}{\pi R^{2}\tau }\right)}^{\frac{1}{2}},$$

The particle velocity induced by the LSW in the Al-Cu alloy is about 1~2 km/s. In this study, a particle velocity of 1.1~1.6 km/s was used to investigate the effects of elevated temperatures on the LSW. The procedures of molecular dynamics simulation can be described as follows:

1. The total energy of the Al-Cu model was decreased to the minimum value by the conjugate gradient method.
2. The whole model was relaxed for 10 ps to make the Al-Cu lattices stable.
3. A particle velocity was loaded on the piston in the direction of [001] orientation for 10 ps.

The loading process can be divided into two parts. The first part is the single shock wave before the wavefront spreads to the boundaries. The other part is a multiple shock wave after the wavefront spreads to the boundaries. The boundaries can lead to rebound shock waves [25]. This study mainly focus on the single shock wave and, thus, the loading time of 2 ps and 4 ps were selected for study.

### 2.3. Characterization of Dislocations

Single-crystal Al is one of the typical FCC metals and, thus, its perfect lattices should be centro-symmetric. Therefore, the centro-symmetry of lattices can be used to recognize the defects in FCC materials. The centro-symmetry parameter (*C*s) is a common character to measure the lattice distortion, which can be written as [26]:

(6) $$C_{S}=\sum_{i=1}^{N/2}{\left|R_{i}+R_{i+\frac{N}{2}}\right|}^{2},$$

where *N* is the number of the nearest-neighbor atoms. *R_i_*, *R_i_*_+*N*/2_ are the vectors from the central atom to its nearest-neighbor atoms. When the nearest-neighbor atoms are symmetric to the central atom, *R_i_* and *R_i_*_+*N*/2_ will offset each other and, thus, *C*s equals zero. When the symmetry of lattices is destroyed by external factors, *R_i_* and *R_i_*_+*N*/2_ cannot offset each other and, thus, *C*s is greater than zero. Since the lattice distortion generated by different lattice defects is significantly different, *C*s can be used to distinguish different types of lattice defects. Since dislocations can generate much more lattice distortion than point defects, the centro-symmetry parameter between 3.5 and 8 Å^2^ was used in this study to recognize the dislocations and stacking faults induced by the LSW.

## 3. Results and Discussion

### 3.1. Propagation Properties of LSW

**Figure 3:**
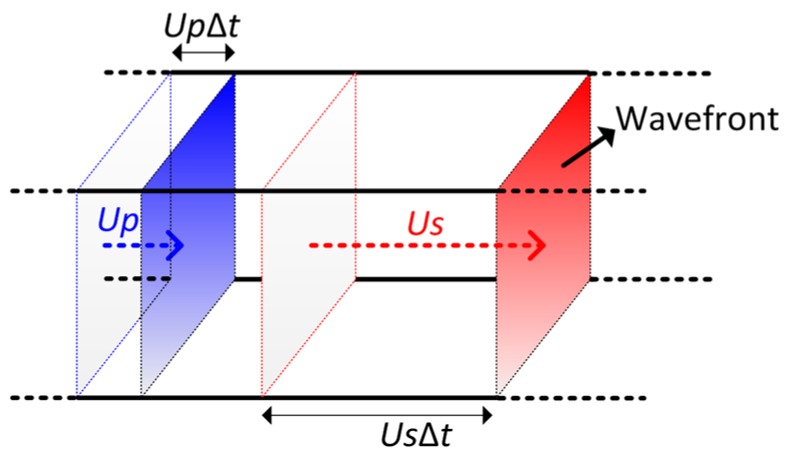
Propagation of a typical shock wave in materials.

The propagation of typical shock waves in materials is shown in Figure 3. A shock velocity *U*_s_ will be induced by a particle velocity *U*_p_. Based on the Hugoniot relation, the shock velocity and the particle velocity are satisfied with the liner relationship as follows [27]:

(7) $$U_{s}=U_{0}+s_{1}U_{p},$$

where *U*_s_ is the shock velocity, *U*_p_ is the particle velocity, *U*_0_ is a constant which is related to the bulk sound speed, and *s*_1_ is a coefficient of the materials. It can be seen that the shock velocity is much greater than the particle velocity.

**Figure 4:**
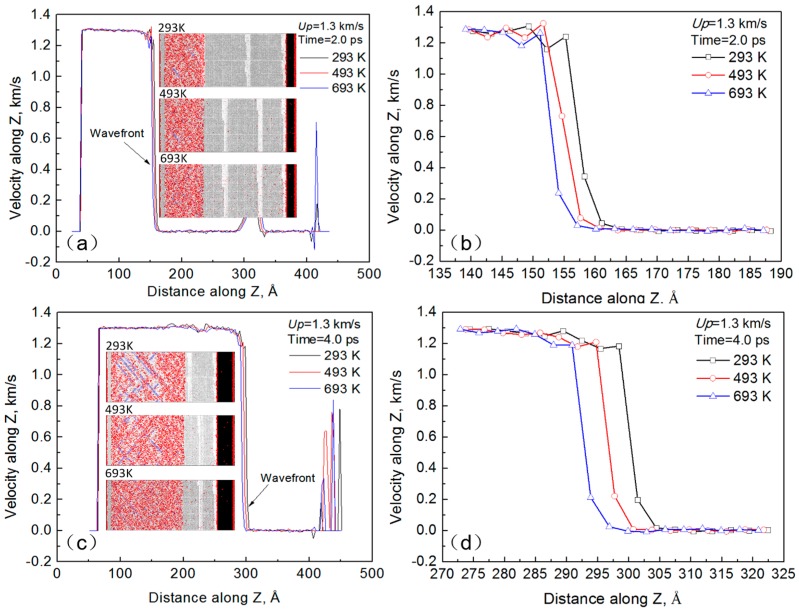
Atomic velocities in shock direction and wavefronts under different temperatures. Loading for (**a**) 2 ps and (**c**) 4 ps. (**b**,**d**) are wavefronts of (**a**,**c**).

After loading for 2 ps and 4 ps, the atomic velocities in shock direction under different temperatures are shown in Figure 4a,c. It can be seen that the atomic velocities are subjected to a sudden change before and after the LSW wavefront. Comparing Figure 4a to Figure 4c, the LSW wavefront rapidly moves along the shock direction as the loading time increases, which indicates that LSW propagates along shock direction rapidly. Moreover, the velocity of piston is much lower than that of LSW wavefront. Coinciding with the Hugoniot relations as shown in Equation (3), the shock velocity is much greater than the particle velocity. In addition, the reasons for the velocity peaks near the back-ends still need further study.

The wavefronts of LSW under different temperatures after 2 ps and 4 ps are shown in Figure 4b,d, respectively. It shows that the wavefront of LSW at 293 K moves slightly faster than that of LSW at 693 K, which indicates that the shock velocity gradually decreases as the temperature increases.

**Figure 5:**
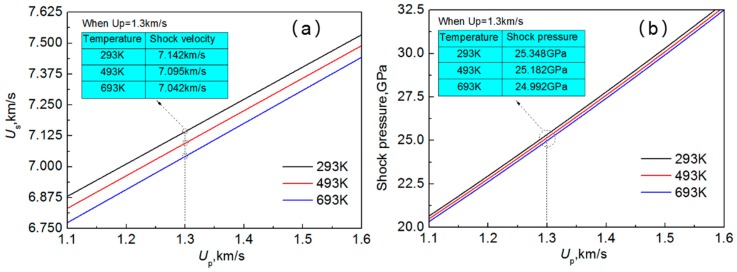
Laser shock properties as a function of the particle velocity under different temperatures. (**a**) Shock velocity; and (**b**) shock pressure.

The shock velocity as a function of the particle velocity under different temperatures is shown in Figure 5a. It shows that the relationship between the particle velocity *U*_p_ and the shock velocity *U*_s_ at different temperatures remains linear relationship, which coincides with Equation (7). Under the temperature of 293 K, 493 K, and 693 K, the parameters of the Hugoniot relations for the Al-Cu alloy are listed in Table 1. As can be seen, the constant *U*_0_ decreases with the increasing temperature, while the other constant *s*_1_ increases with the increasing temperature. It can be inferred that the bulk speed of sound decreases as the temperature increases. Moreover, when the particle velocity is 1.3 km/s, the shock velocity under the temperatures of 293 K, 493 K, and 693 K are 7.142 km/s, 7.095 km/s, and 7.042 km/s, respectively. This indicates that the shock velocity of LSW decreases with the increase of temperatures.

Moreover, based on the Hugoniot relations, the the shock pressure *P* induced by the particle velocity *U*_p_ can be written as:

(8) $$P=\rho U_{s}U_{p},$$

where *P* is the shock pressure in the materials and ρ is the density of materials. The density of the Al-Cu alloy is about 2.73 g/cm^3^.

The shock pressure as a function of the particle velocity under different temperatures is shown in Figure 5b. It can be seen that when the particle velocity is 1.3 km/s, the shock pressure induced by LSW at 293 K, 493 K, and 693 K is 25.348 GPa, 25.182 GPa, and 24.992 GPa, respectively. The results indicate that the shock pressure decreases with the increase of temperatures, which is consistent with Equation (8).

### 3.2. Elastic and Plastic Waves Generated by LSW in the [001] Direction

**Figure 6:**
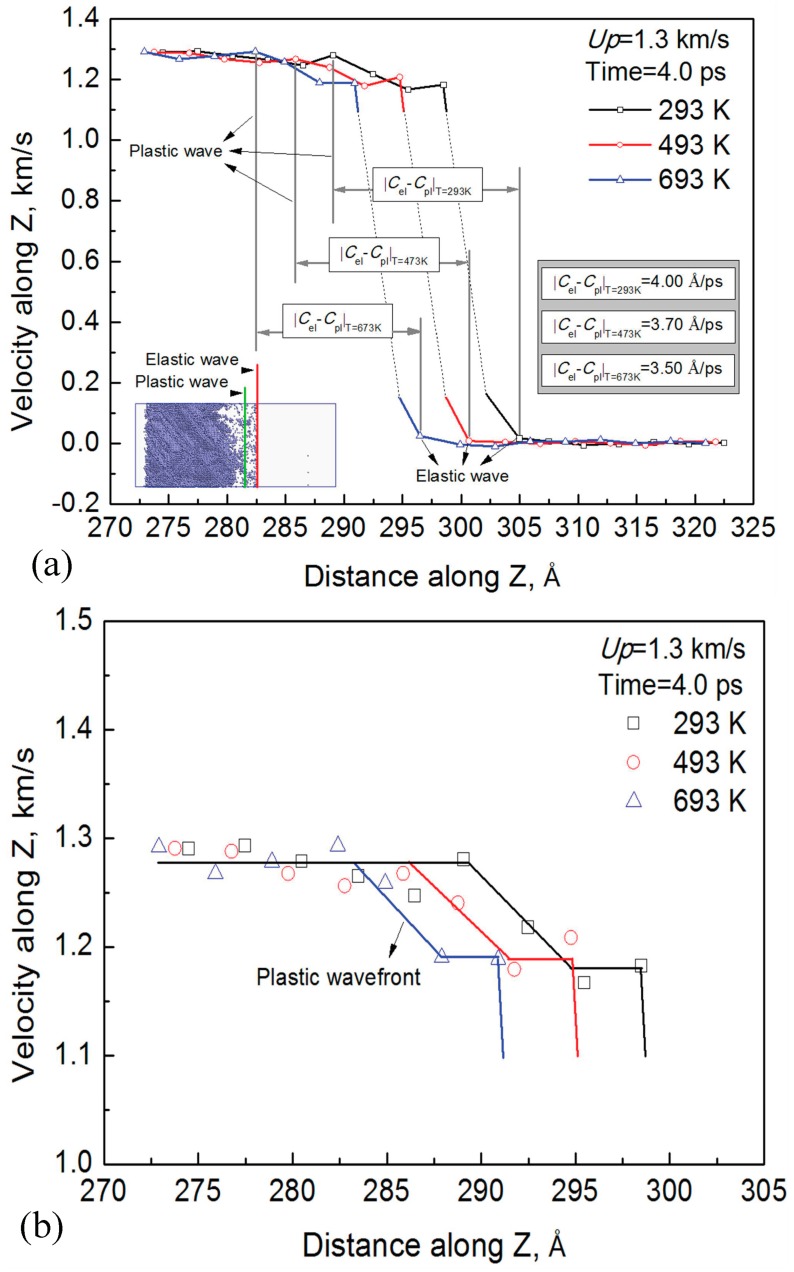
Elastic and plastic waves induced by the LSW in the [001] direction. (**b**) is the local magnification of (**a**).

In order to analyze the elastic and plastic waves induced by LSW in the [001] direction, the laser wavefronts in the shock direction were further studied and the results are shown in Figure 6. It can be found in Figure 6a that there are two different wavefronts at all temperatures. This means the LSW can be divided into an elastic wave and plastic wave in the shock direction, which can also be proved by the stacking faults induced by the LSW under 293 K. There is a short distance from the stacking faults to the wavefront, which indicates that the velocity of the plastic wave is slightly lower than that of the elastic wave. Moreover, the elastic wave velocity decreases with increasing temperature. Figure 6b is the local magnification of the plastic wavefronts in Figure 6a. This indicates that the plastic wave velocity also decreases with the increasing temperatures.

In addition, it can be seen in Figure 6a that the difference between the velocity of the plastic wave and the elastic wave |*C*_el_ − *C*_pl_| also decreases as temperature increases. Since the properties along certain lattice directions are almost the same, the Al-Cu crystals along one certain direction can be treated as an isotropic solid. Therefore, based on the research of Berth [28], the velocity of the elastic wave *C*_el_ and the velocity of the plastic wave *C*_pl_ can be written as:

(9) $$C_{\mathrm{el}}=\sqrt{\frac{\lambda +2\mu }{\rho }}\;,$$

(10) $$C_{\mathrm{pl}}=\sqrt{\frac{\lambda +\frac{2\mu }{3}}{\rho }}\;,$$

where λ and μ are Lame constants, and ρ is the material’s density. Lame constants can be calculated by:

(11) $$\lambda =\frac{E\nu }{\left(1+\nu \right)\left(1-2\nu \right)},$$

(12) $$\mu =\frac{E}{2\left(1+\nu \right)},$$

where *E* is the elastic modulus and ν is the Poisson ratio. Based on the following equation:

(13) $$E=E_{0}\left(1-\beta T\right)$$

where *E*_0_ is the initial elastic modulus, *T* is the treating temperature, and β is a constant related to the coefficient of thermal expansion. Therefore, *C*_el_ and *C*_pl_ can be written as:

(14) $$C_{\mathrm{el}}=\sqrt{\frac{1-\nu }{\rho \left(1+\nu \right)\left(1-2\nu \right)}}\;\cdot \sqrt{E_{0}\left(1-\beta T\right)},$$

(15) $$C_{\mathrm{pl}}=\sqrt{\frac{1+\nu }{3\rho \left(1+\nu \right)\left(1-2\nu \right)}}\;\cdot \sqrt{E_{0}\left(1-\beta T\right)},$$

It can be seen in Equations (14) and (15) that the velocity of the elastic wave *C*_el_ and the plastic wave *C*_pl_ both decrease with the increasing treatment temperature, which is similar to the results in Figure 6a,b.

Moreover, it can be obtained from Equations (14) and (15) that:

(16) $$C_{\mathrm{el}}-C_{\mathrm{pl}}=\alpha \sqrt{E_{0}\left(1-\beta T\right)},$$

where$\;\alpha =\sqrt{\frac{1-\nu }{\rho \left(1+\nu \right)\left(1-2\nu \right)}}-\sqrt{\frac{1+\nu }{3\rho \left(1+\nu \right)\left(1-2\nu \right)}}$. It is shown in Equation (16) that the difference between the velocity of the elastic wave and the plastic wave decreases with the increasing temperatures, which coincides with Figure 6a.

### 3.3. Dislocations Induced by LSW

**Figure 7:**
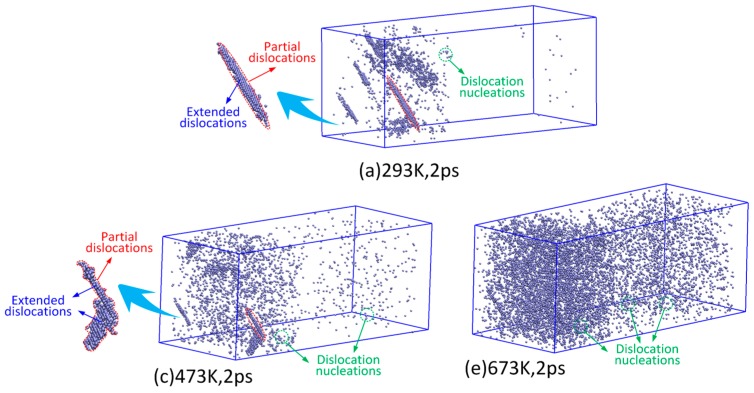
Evolution of dislocation atoms induced by the laser shock wave at 2 ps under different temperatures; (**a**) 293 K; (**b**) 473 K; and (**c**) 673 K.

The evolution of dislocation atoms generated by LSW at 2 ps is shown in Figure 7. As can be seen from the Figure 7a, when the temperature is 293 K, dislocations induced by the LSW can be divided into two parts: dislocation nucleations and extended dislocations. The extended dislocations are the two partial dislocations connected by stacking faults. Dislocation nucleations are mainly generated by the point defects, especially the vacancies. Since the {111} planes are the close-packed plane of Al-Cu alloys, stacking faults first appear in the {111} planes and, thus, extended dislocations mainly originate in the {111} planes under laser shock pressure. Similar to the LSW at 293 K, the plastic strain induced by the LSW at 473 K is mainly generated by dislocation nucleations and extended dislocations, as shown in Figure 7b. However, the extended dislocations are decreased while the dislocation nucleations are increased by the increasing temperature. Different from the LSW at 293 K and 473 K, there are few extended dislocations generated by the LSW at 673 K, as shown in Figure 7c. However, dislocation nucleations induced by the LSW at 673 K are much greater than that induced by the LSW at 293 K and 473 K. The plastic strain is mainly generated by dislocation nucleations for the LSW at 673 K.

**Figure 8:**
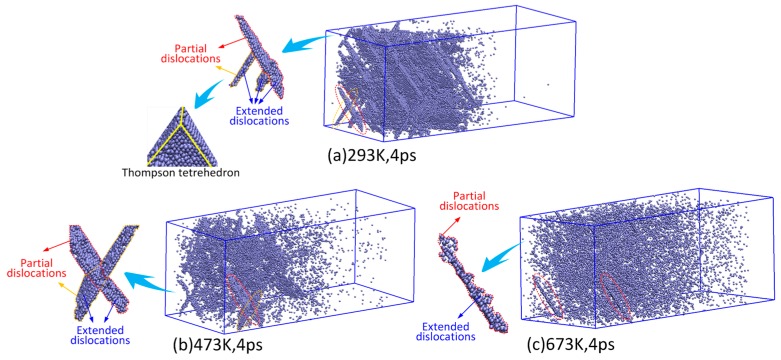
Evolution of the dislocation atoms induced by the laser shock wave at 4 ps under different temperatures; (**a**) 293 K; (**b**) 473 K; and (**c**) 673 K.

The evolution of the dislocation atoms generated by the LSW at 4 ps is shown in Figure 8. It can be seen in Figure 8a that many partial stacking faults in extended dislocations developed into complete stacking faults in the {111} planes and, thus, there are many Thompson tetrehedrons generated by the LSW at 293 K. As shown in Figure 8b, there are few complete stacking faults generated by the LSW at 473 K although the extended dislocations also develop with loading time. Thus, there are no Thompson tetrehedrons generated by LSW at 473 K. When the temperature increases to 673 K, there are only very small amounts of extended dislocations, as shown in Figure 8c. The number of dislocation nucleations is further increased by elevated temperatures. However, because there are not many stacking faults under elevated temperatures, the dislocations generated by point defects are dispersed in material with a small scale. The results above show that elevated temperature has significant effects on the formation and development of dislocations generated by the LSW.

Compared with Figure 7 and Figure 8, it can also be found that the loading time also has significant effects on the dislocations induced by the LSW. Based on the mechanics of materials, the strain rate $\dot{\varepsilon }$can be written as:

(17) $$\dot{\varepsilon }=\frac{\varepsilon }{\Delta t},$$

where ε is the strain and Δ*t* is the time interval. Combined with the process of the LSW, Equation (17) can be written as:

(18) $$\dot{\varepsilon }=\frac{\varepsilon }{\Delta t}=\frac{\Delta S}{\Delta t}=U_{p},$$

where Δ*S* is the displacement of piston. Therefore, the strain during LSW can be written as:

(19) $$\varepsilon =\dot{\varepsilon }\Delta t=U_{p}\Delta t,$$

In this study, the particle velocity *U*_p_ is constant in time and, thus, the strain increases with the increasing loading time. Since the dislocations always increase with the increasing strain, the dislocations can also be increased by the loading time of the LSW.

**Figure 9:**
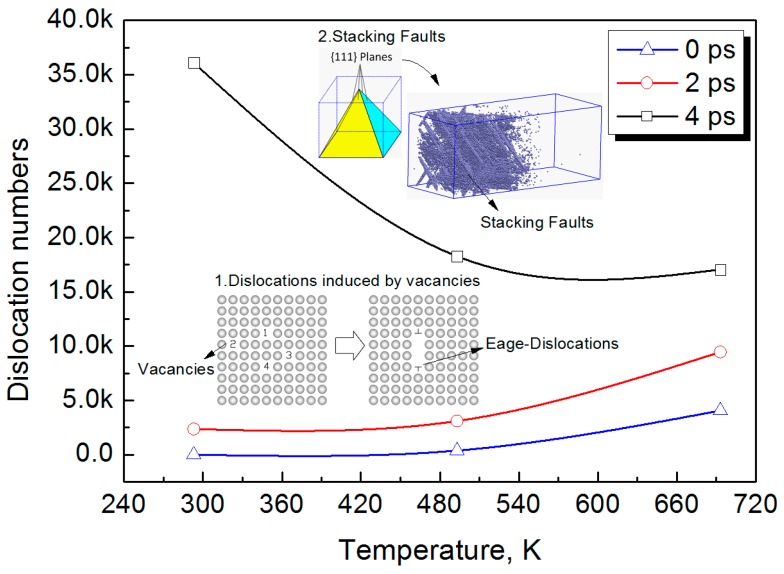
Number of dislocation atoms induced by the laser shock wave under different temperatures.

The number of dislocations of atoms induced by the LSW under different temperatures is shown in Figure 9. It can be seen that the number of dislocation atoms induced by the LSW increases as the loading time increases. On the other hand, the number of dislocation atoms induced by LSW increases with the increasing temperature before 2 ps, but it decreases with the increasing temperature after 2 ps. When the loading time is 4 ps, the number of dislocation atoms induced by the LSW at 293 K is 1.97 and 2.12 times as many as that obtained at 473 K and 673 K, respectively. The results above indicate that the dislocation mechanism is subjected to a change before and after 2 ps.

**Figure 10:**
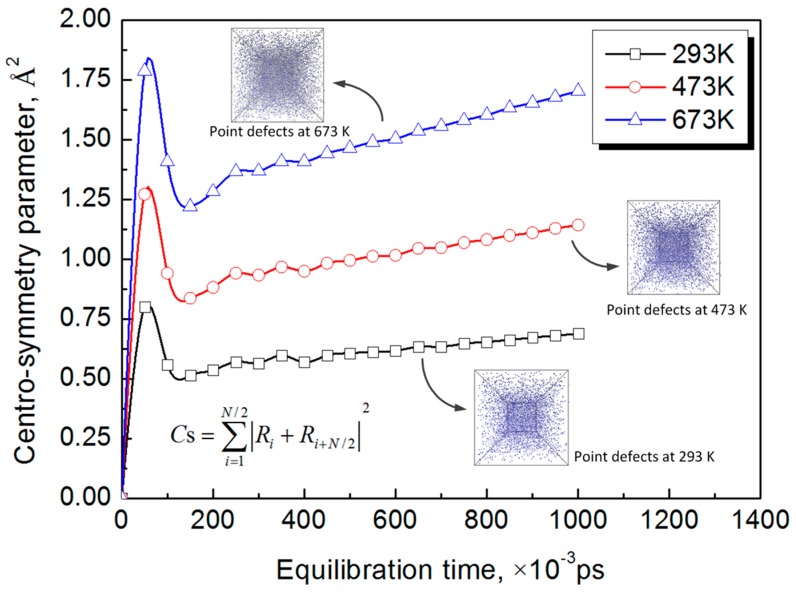
Centro-symmetry parameter as a function of equilibration time under different temperatures.

**Figure 11:**
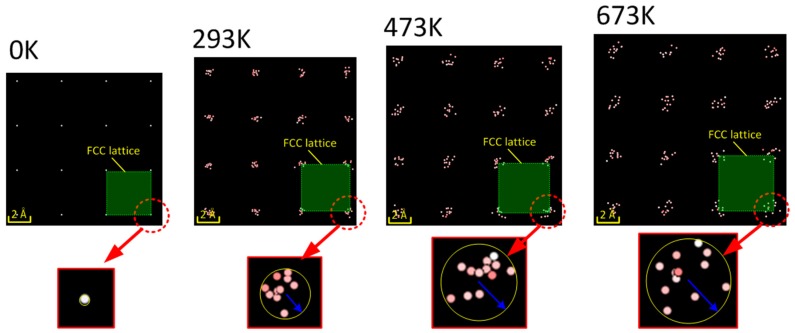
Atoms in the lattices of Al-Cu alloy under different temperatures.

Before 2 ps, there are not many extended dislocations and stacking faults formed in the Al-Cu alloy because of the low strain. Thus, the resistance of dislocation slip is small and, thus, the dislocations increase slowly under the pressure of the LSW. The major development mechanism of dislocations are dislocation nucleations generated by point defects. The centro-symmetry parameter as a function of equilibration time under different temperatures can be seen in Figure 10. It is shown that *C*_s_ increases with the increasing temperature, which indicates that there are more lattice defects in the material under elevated temperatures. This is because the elevated temperature decreases the attractive forces between the atoms and increases the atomic spacing, as shown in Figure 11. The increasing atomic spacing make the atoms much easier to break away from the lattice and, thus, form more point defects. The point defects, such as vacancies, will cluster, collapse, and then form dislocation nucleations [29]. Moreover, because the point defects increase with the increasing temperatures, the dislocation nucleations and dislocation atoms increase as the temperature increases.

**Figure 12:**
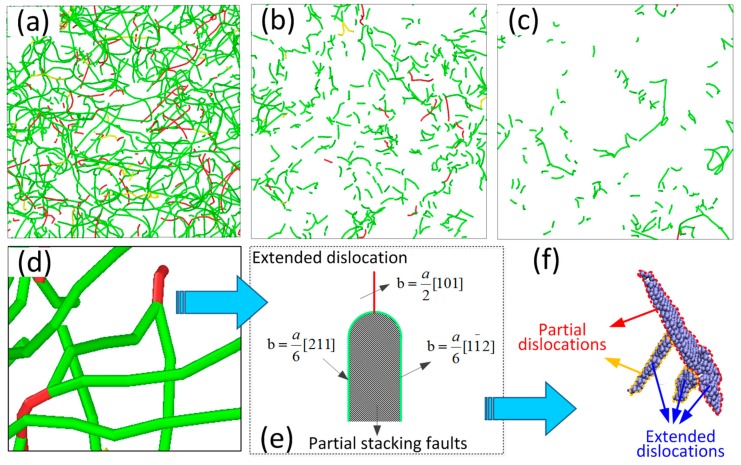
Analysis for the dislocations generated by LSW under different temperatures. (**a**) 293 K, 4 ps; (**b**) 473 K, 4 ps; (**c**) 673 K, 4 ps; (**d**) extended dislocations; (**e**) Burgers vectors of extended dislocation; and (**f**) extended dislocation atoms.

After 2 ps, the extended dislocations significantly increase under the LSW due to the increasing strain. The dislocation analysis (DXA) solver in Ovito software (Version OVITO 2.8.1, Darmstadt University of Technology, Darmstadt, Germany) was used in this study to investigate the development of extended dislocations. The results can be seen in Figure 12. Figure 12a–c are the dislocations in the (001) plane generated by the LSW under 293 K, 473 K, and 673 K, respectively. It can be seen in Figure 12a that there are many extended dislocations which have the structure as shown in Figure 12d. Based on dislocation theory [29], the structure of the extended dislocations can be shown in Figure 12e. A complete dislocation with a Burgers vector of $\frac{a}{2}\;\left[101\right]$ is split into two Shockley partial dislocations with Burgers vectors of $\frac{a}{6}\;\left[211\right]$ and $\frac{a}{6}\;\left[1\bar{1}2\right]$. The space between two Shockley partial dislocations is filled with partial stacking faults, as show in Figure 12f. The rapid formation and development of extended dislocations can effectively increase the resistance of dislocation slip and, thus, promote further nucleation and development of dislocations. Thus, the dislocation atoms increase significantly after 2 ps. In addition, compared with Figure 12a, extended dislocations are significantly decreased by the elevated temperatures as shown in Figure 12b,c. The reason is that elevated temperatures can decrease the stacking faults and thus make extended dislocation difficult to form and develop.

Moreover, because the extended dislocations significantly decrease with the increasing temperature, it can be seen in Figure 9 that the number of dislocation atoms in Al-Cu alloy decreases with the increasing temperatures when the loading time reaches 4 ps.

## 4. Conclusions

The thermal effects on the propagation properties and dislocation evolution induced by LSW in an Al-Cu alloy were studied by the molecular dynamics method. The conclusions can be described as follows:

(1) Within the strain rate of LSW, the shock velocity and shock pressure both decrease with the increasing temperatures.
(2) LSW can be divided into an elastic wave and plastic wave in the [001] direction. The velocity of the elastic wave and plastic wave both decrease with the increasing treatment temperature. Moreover, the difference between the velocity of the elastic wave and plastic wave decreases as the temperature increases.
(3) The dislocation atoms induced by LSW increases with the increasing temperature before 2 ps, while it decreases with the increasing temperature after 2 ps. The reasons for the results are related to the formation and evolution of extended dislocations. The mechanism of dislocation proliferation is due to dislocation nucleations generated by point defects before 2 ps, while extended dislocation developed after 2 ps.

## Figures and Tables

**Table 1:** Parameters in Hugoniot relations for laser shock wave under different temperatures.

Temperature	*U*_0_ (km/s)	*S*_1_
293 K	5.4422	1.3077
493 K	5.3841	1.3163
693 K	5.3021	1.3383
